# A118 THE IMPACT OF HOMELESSNESS ON ENDOSCOPY UTILIZATION, SURGICAL TREATMENT, AND POST-OPERATIVE COMPLICATIONS AMONG HOSPITALIZED PATIENTS WITH CROHN’S DISEASE

**DOI:** 10.1093/jcag/gwad061.118

**Published:** 2024-02-14

**Authors:** M Youssef, T Mungle, V Charu, P Okafor

**Affiliations:** Internal Medicine, University of Toronto, Toronto, ON, Canada; Stanford Center for Biomedical Informatics Research, Stanford, CA; Stanford University Department of Pathology, Stanford, CA; Mayo Clinic Division of Gastroenterology and Hepatology, Jacksonville, FL

## Abstract

**Background:**

Homelessness is an important public health issue with a prevalence around 4.2% in the United States. Homelessness has been associated with lack of access to healthcare services, poor quality of care, and negative health outcomes. However, there is limited data on clinical outcomes among homeless individuals with Crohn’s disease (CD).

**Aims:**

In this study, we examine inpatient outcomes including endoscopy utilization, surgical treatment, and post-operative complications among homeless patients with CD.

**Methods:**

The Healthcare Utilization Project (HCUP) State Inpatient Databases from New York, Arizona, Massachusetts and Florida for 2013 and 2014 were used to identify adults ≥18 years admitted with CD identified by International Classification of Diseases, Ninth Revision codes. Homeless patients were then identified, and propensity score matched using a one-to-ten greedy nearest-neighbor approach to non-homeless patients to balance the distribution of baseline covariates. Differences in outcomes including endoscopy utilization, surgical treatment and post-operative complications were reported as odds ratios (ORs) and 95% confidence intervals (CI).

**Results:**

A total of 70,457 CD hospitalizations were identified, of which 469 (0.6%) were associated with homelessness. We matched 440 homeless patients with 4,400 housed patients with CD. Homeless individuals had a higher proportion of males (p=0.002), tended to be older (p=0.035), and had more comorbidities than non-homeless individuals. There was no difference in mortality between non-homeless and homeless patients (OR 1.6, 95% CI 0.72-3.57). Endoscopy utilization was comparable between non-homeless and homeless patients with CD including gastroscopy (OR 0.71, 95% CI 0.40-1.18), colonoscopy (OR 0.80, 95% CI 0.31-1.69), and sigmoidoscopy (OR 0.37, 95% CI 0.02-1.74) [Figure 1]. Surgical treatment was also comparable, except for lower rates of bowel resection among homeless patients (OR 0.55, 95% CI: 0.29-0.93). The two groups also had similar rates of post-operative complications including pulmonary, cardiovascular, gastrointestinal, and infectious complications (Figure 1).

**Conclusions:**

Homeless patients with CD have comparable endoscopy utilization and surgical treatment as non-homeless patients, except for lower bowel resection rates. Postoperative complications are also similar between the two groups. Future studies are needed to better understand the impact of homelessness on health outcomes and healthcare service utilization among patients with CD.

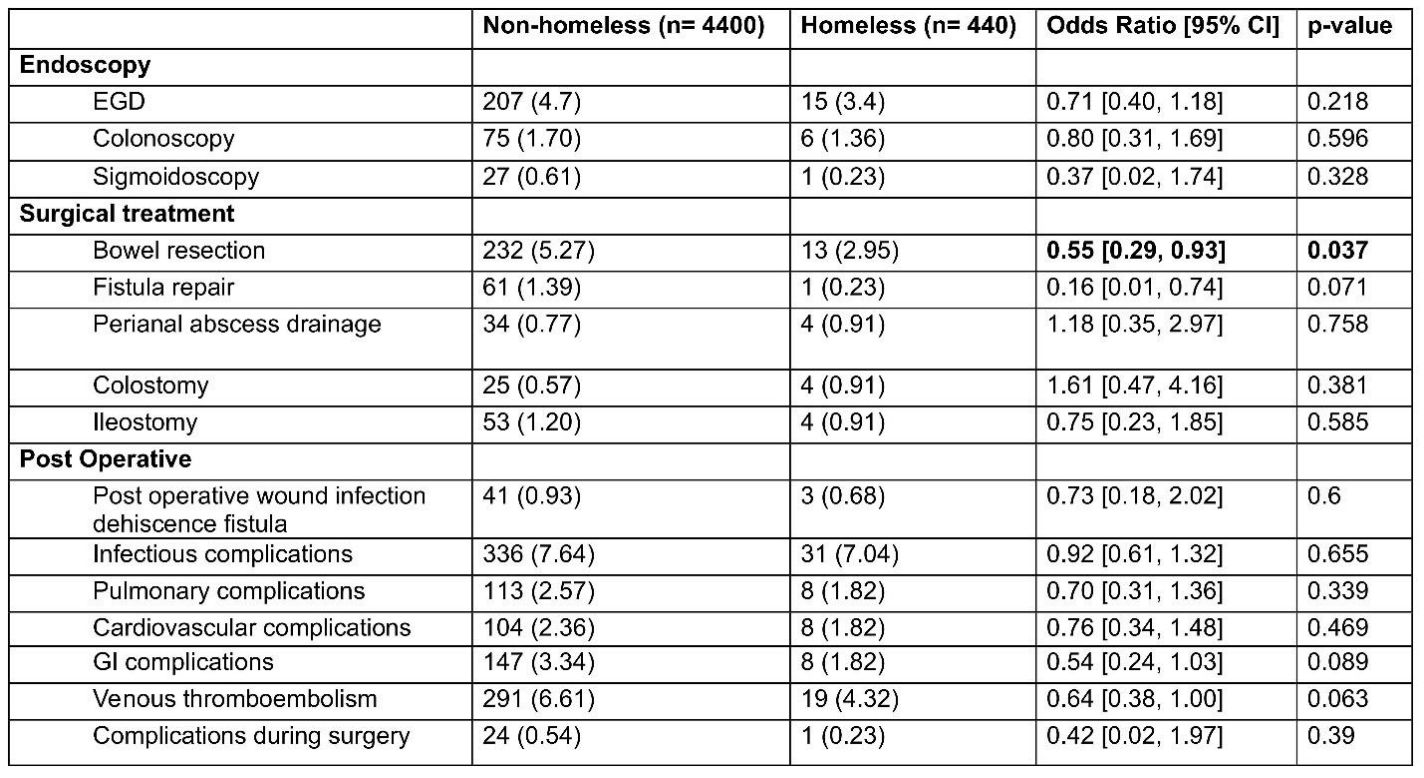

Figure 1. Endoscopy utilization, surgical treatment, and post-operative complications among homeless and non-homeless individuals using 1:10 propensity matching

**Funding Agencies:**

None

